# Hypoxia imaging with ^18^F-FAZA PET/CT predicts radiotherapy response in esophageal adenocarcinoma xenografts

**DOI:** 10.1186/s13014-018-0984-3

**Published:** 2018-03-07

**Authors:** Elodie Melsens, Elly De Vlieghere, Benedicte Descamps, Christian Vanhove, Ken Kersemans, Filip De Vos, Ingeborg Goethals, Boudewijn Brans, Olivier De Wever, Wim Ceelen, Piet Pattyn

**Affiliations:** 1 Laboratory of Experimental Surgery, Department of Gastro- Intestinal Surgery, Ghent University Hospital, De Pintelaan 185, B-9000 Ghent, Belgium; 2 Laboratory of Experimental Cancer Research, Department of Radiation Oncology and Experimental Cancer Research, Ghent University, Ghent, Belgium; 3 Infinity (IBiTech-MEDISIP), Department of Electronics and Information Systems, Ghent University, Ghent, Belgium; 4 Department of Nuclear Medicine, Ghent University Hospital, Ghent, Belgium; 5 Department of Pharmaceutical Analysis, Ghent University, Ghent, Belgium; 6 Cancer Research Institute Ghent (CRIG), Ghent University, Ghent, Belgium

**Keywords:** Radioresistance, ^18^F-FAZA pet/CT, Tumor hypoxia, Predictive biomarker, Esophageal adenocarcinoma xenografts, Nimorazole

## Abstract

**Background:**

Esophageal cancer is an aggressive disease with poor survival rates. A more patient-tailored approach based on predictive biomarkers could improve outcome. We aimed to predict radiotherapy (RT) response by imaging tumor hypoxia with ^18^F-FAZA PET/CT in an esophageal adenocarcinoma (EAC) mouse model. Additionally, we investigated the radiosensitizing effect of the hypoxia modifier nimorazole in vitro and in vivo.

**Methods:**

In vitro MTS cell proliferation assays (OACM5 1.C SC1, human EAC cell line) were performed under normoxic and hypoxic (< 1%) conditions: control (100 μL PBS), nimorazole, irradiation (5, 10 or 20 Gy) with or without nimorazole. In vivo*,* subcutaneous xenografts were induced in nude mice (OACM5 1.C SC1). Treatment was given daily for 5 consecutive days: (A) control (600 μl NaCl 0.9% intraperitoneally (IP)) (*N* = 5, *n* = 7), (B) RT (5 Gy/d) (*N* = 11, *n* = 20), (C) combination (nimorazole (200 mg/kg/d IP) 30 min before RT) (*N* = 13, *n* = 21). N = number of mice, n = number of tumors. ^18^F-FAZA PET/CT was performed before treatment and tumor to background (T/B) ratios were calculated. Relative tumor growth was calculated and tumor sections were examined histologically (hypoxia, proliferation).

**Results:**

A T/B ≥ 3.59 on pre-treatment ^18^F-FAZA PET/CT was predictive for worse RT response (sensitivity 92.3%, specificity 71.4%). Radiation was less effective in hypoxic tumors (T/B ≥ 3.59) compared to normoxic tumors (T/B < 3.59) (*P* = 0.0025). In vitro, pre-treatment with nimorazole significantly decreased hypoxic radioresistance (*P* < 0.01) while in vivo, nimorazole enhanced the efficacy of RT to suppress cancer cell proliferation in hypoxic tumor areas (Ki67, *P* = 0.064), but did not affect macroscopic tumor growth.

**Conclusions:**

Tumor tissue hypoxia as measured with ^18^F-FAZA PET/CT is predictive for RT response in an EAC xenograft model. The radiosensitizing effect of nimorazole was questionable and requires further investigation.

**Electronic supplementary material:**

The online version of this article (10.1186/s13014-018-0984-3) contains supplementary material, which is available to authorized users.

## Background

Esophageal cancer patients are mostly diagnosed in a locally advanced stage and treated with neoadjuvant chemoradiation followed by surgery [1]. Prognosis is poor and response to treatment is highly variable [2]. Identification of predictive imaging biomarkers is an important challenge.

Tumor hypoxia is an attractive predictive factor as it has been correlated with chemoresistance, radioresistance, invasiveness, propensity to metastasize, genomic instability and worse prognosis in different solid tumors [3].

Also in esophageal cancer, hypoxia has been correlated with worse outcomes. Histologic examination of carbonic anhydrase 9 (CAIX) and hypoxia-inducible factor 1-alpha (HIF-1α), two factors that are overexpressed in hypoxic conditions, were correlated with worse outcomes and hypoxia imaging with ^18^F-FETNIM (fluoroerythronitroimidazole) positron emission tomography (PET) showed that tracer uptake might be predictive for treatment response in esophageal cancer [4–7].

PET-based hypoxia imaging is one of the most studied hypoxia detection methods with clinical applicability. Over the years, different tracers have been studied and have been proven to have predictive or prognostic value (^18^F-FMISO (fluoromisonidazole) [8], ^18^F-FAZA (fluoroazomycin arabinoside) [9], ^18^F-FETNIM [6], ^18^F-EF5 (pentafluoropropylacetamide) [10, 11]). Here, ^18^F-FAZA PET/CT was used to image tumor hypoxia and investigate its predictive potential in esophageal cancer. ^18^F-FAZA is a second generation 2-nitroimidazole that has been shown to be hypoxia specific and reproducible [12]. It has superior pharmacokinetics compared to ^18^F-FMISO, resulting in a better tumor-to-background ratio [13]. The tracer entrapment is based on a reduction of the NO2-group followed by continued reduction under hypoxic conditions and eventually covalent binding to intracellular macromolecules [14]. This non-invasive technique provides a 3-dimensional image of the hypoxia distribution and can be repeated over time, which allows follow-up [14]. ^18^F-FAZA PET is a promising tracer that already showed to be predictive for treatment response in preclinical models of rhabdomyosarcoma and breast carcinoma [15, 16]. Clinically, FAZA imaging has been studied in non-small cell lung cancer [17] and head and neck squamous cell cancer [18, 19], while trials are ongoing in rectal, lung, cervix, and prostate carcinoma (ClinicalTrials.gov: NCT02624115, NCT02701699, NCT01989364, NCT01567800).

Additionally, we investigated whether nimorazole could enhance radiation response in hypoxic conditions. It is a 5-nitroimidazole that mimics oxygen in the radiobiological process by promoting fixation of free radicals [20]. Nimorazole is easy applicable, has few side effects and is already part of daily practice in Denmark for HNSCC patients [21] (DAHANCA guidelines).

In summary, this study investigated the predictive value of ^18^F-FAZA PET/CT for hypoxia-induced radioresistance in EAC xenografts and the radiosensitizing effect of nimorazole.

## Methods

### Cell line

OACM5 1.C SC1 was established through in vivo selection from the parental cell line OACM5 1.C, a human esophageal adenocarcinoma (EAC) cell line, as described previously [22] and was authenticated by STR-based DNA-profiling. Cells were cultured at 37 °C in 5% CO2 humidified atmosphere in RPMI 1640 Medium supplemented with GlutaMAX™-I (Life Technologies), 10% fetal bovine serum and penicillin-streptomycin.

### MTS assay

Hypoxic radioresistance and the radiosensitizing effect of nimorazole were first quantified in vitro with an MTS (3-(4,5-dimethylthiazol-2-yl)-5-(3-carboxymethoxyphenyl)-2-(4-sulfophenyl)-2H-tetrazolium) assay. Cells (8 × 10^5^ per T25 flask) were incubated overnight at normoxic (5% CO_2_ in air) or hypoxic (Anaerobic Work Station, Baker Ruskinn, 80% N_2_, 10% CO_2_, 10% H_2_) conditions. Treatment was given 24 h after seeding: control (100 μL PBS); nimorazole (0.2 mg/mL in PBS); RT (5, 10 or 20 Gy) with or without nimorazole. The metabolic activity of cells was analyzed 72 h post-treatment. A solution of a tetrazolium compound (MTS, CellTiter 96® Aqueous MTS Reagent Powder (Promega)) and an electron coupling reagent phenazine methosulfate (PMS) was added to each T25 flask (1 mL) and was incubated for 90 min (37 °C and 5% CO_2_). Absorbance was measured with Paradigm (490 nm) (SPECTRAMax Paradigm, Molecular Devices, USA). Cell viabilities were calculated relative to controls (0 Gy = 100% cell viability). (*n* = 3 × 2).

### Animals and tumor model

Animal experiments were approved by the Animal Ethical Committee of the Ghent University (ECD 14/82) and were performed in accordance with the EU Directive 2010/63/EU. OACM5 1.C SC1 cells (3 × 10^6^ in 100 μl of Matrigel) were injected subcutaneously in both hind legs of athymic male mice (5 weeks of age, Foxn1nu, Envigo, the Netherlands). Tumors were grown for 7 weeks. Tumors with a minimum volume of 150 mm^3^ were included. One day post-treatment, mice were euthanized under anesthesia by cervical dislocation. Inhalation anesthesia with isoflurane (Abbott, Belgium) was used, 5% induction, 1.5% maintenance, 0.3 L/min.

### Treatment

Treatment was given daily for 5 consecutive days: (A) control (600 μl NaCl 0.9% intraperitoneally (IP)) (*N* = 5, *n* = 7), (B) RT (5 Gy/d) (*N* = 11, *n* = 20), (C) combination (nimorazole (200 mg/kg/d IP) 30 min before RT) (*N* = 13, *n* = 21). N = number of mice, n = number of tumors. Nimorazole (Adooq Bioscience LLC, USA) was dissolved in NaCl 0.9% at 10 mg/mL on the day of administration. The dosage and timing was according to previous literature [23]. Because nimorazole acts as a pure radiosensitizer at this dosage, no nimorazole monotherapy group was included *(*See in vitro results and [23]). Tumor nodules were measured daily with calipers and volumes were calculated according to the following formula: *V* = (*length* × *width*)^3/2^ × *π*/6. Relative tumor growth (RTG) was calculated as the ratio of the volume at the day of euthanasia to the volume before treatment.

### Radiotherapy

RT was applied using the small animal radiation research platform (SARRP). The voltage of the X-ray source was fixed at 220 kV with a tube current of 13 mA, emitted from the 3 mm focal spot, filtered by a copper filter of 0.15 mm. For in vitro experiments, a vertical radiation beam of 10 × 10 cm^2^ was used. Single doses of 5, 10 or 20 Gy were administered. For in vivo experiments, a pair of parallel-opposed (anterior-posterior) radiation beams of 10 × 10 mm^2^ were used. Mice were anesthetized and positioned on the bed of the SARRP. Guided by lasers, the bed was moved to position tumors at the isocenter of the beam. To allow parallel-opposed beam irradiations, mice were turned around when half of the dose was given. Tumors were irradiated 5 consecutive days, 5 Gy/day.

### ^18^F-FAZA pet-CT

The radiosynthesis of ^18^F-FAZA was performed on a Synthra RNplus module (Synthra GmbH, Hamburg, Germany) using a fully automated procedure that was based on standard procedures [24, 25]. The precursor for the radiosynthesis, 1-(2,3-diacetyl-5-tosyl-(α-d-arabinofuranosyl)-2-nitroimidazole, was purchased from ABX GmbH (Radeberg, Germany) and all other required reagents and solvents were acquired from Sigma-Aldrich (Overijse, Belgium).

^18^F-FAZA PET/CT was performed one day before treatment. Mice were anesthetized and 37.0±1.9 MBq of ^18^F-FAZA was injected in the tail vein. Three hours after injection and under anesthesia a static PET/CT was performed. The animals were positioned on a heated bed of a small animal PET/CT scanner (TriFoil Imaging, Triumph II, Northridge, CA, USA). A 30 min PET scan was acquired in list mode, with a 75-mm axial field-of-view and a 1.3-mm spatial resolution. On the same scanner and without moving the animal, a CT scan was performed. CT projection data were acquired using the following parameters: 256 projections, detector pixel size 50 μm, focal spot size 100 μm, tube voltage 50 kV, tube current 640 μA, and a field-of-view of 90 mm. The acquired PET images were reconstructed into a 200x200x64 matrix by a 2D maximum likelihood expectation maximization (MLEM) algorithm (LabPET Version 1.12.1, TriFoil Imaging®, Northridge, CA) using 50 iterations and a voxel size of 0.5 × 0.5 × 1.175 mm^3^ (x, y, z). CT images were analytically reconstructed using a filtered back projection reconstruction algorithm (Cobra Version 7.3.4, Exxim Computing Corporation, Pleasanton, CA) into a 256x256x512 matrix with 200 μm isotropic voxel size. Each resultant CT image is inherently co-registered with the corresponding PET scan. PET and CT images were imported into A Medical Image Data Examiner (AMIDE) [26], where tumor-to-background (T/B) ratios were calculated as the mean tumor uptake divided by the background activity. Mean tumor uptake (Bq/mL) was quantified in a volume-of-interest that was semi-automatically delineated as the activity > 40% of the maximum activity using the 3D-isocontour tool, similar to Tran et al. [27], and a sphere with radius 1.5 mm was delineated in the foreleg muscle as background tissue.

### Tumor samples and histology

Consecutive 5 μm sections of FFPE tumors were prepared. H&E staining was performed and necrotic areas were excluded for further analysis. The hypoxia marker pimonidazole, administered 1 h before sacrifice (60 mg/kg, IP, Hypoxyprobe, USA), was stained with Hypoxyprobe anti-pimonidazole Ab (HP1–100 Kit)(1/50). Ki67 staining was performed with anti-Ki67 Ab ([SP6] Abcam 16,667)(1/100) and proliferation indices (fraction of Ki67+ cells/total cells) were calculated in normoxic and hypoxic regions, according to pimonidazole staining on consecutive sections (3 × 2 hotspots/tumor) (ImageJ (ImmunoRatio)). Microscopy was performed with a light microscope (ColorView I, BX43F, Olympus, Japan).

### Statistical methods

Statistical analysis was performed with GraphPad Prism6 (Graphpad Software, Inc.: La Jolla, USA). Data was tested for normality (Shapiro-Wilk) and analyzed with the Mann-Whitney U test (non-parametric) or t-test (parametric). ID50 values of the MTS-assay were calculated with non-linear regression analysis (log(inhibitor) vs. normalized response). Oxygen Enhancement Ratio (OER = Radiation dose hypoxia/normoxia) and Sensitizer Enhancement Ratio (SER = Radiation dose hypoxia/hypoxia with nimorazole) were calculated. The cut-off T/B ratio to predict treatment response was determined with ROC-analysis. *P*-values < 0.05 were considered statistically significant and abbreviated as * = *P* < 0.05, ** = *P* < 0.01, *** = P 0.001, **** = *P* < 0.0001.

## Results

### ^18^F-FAZA PET/CT as predictive biomarker

**Fig. 1:**
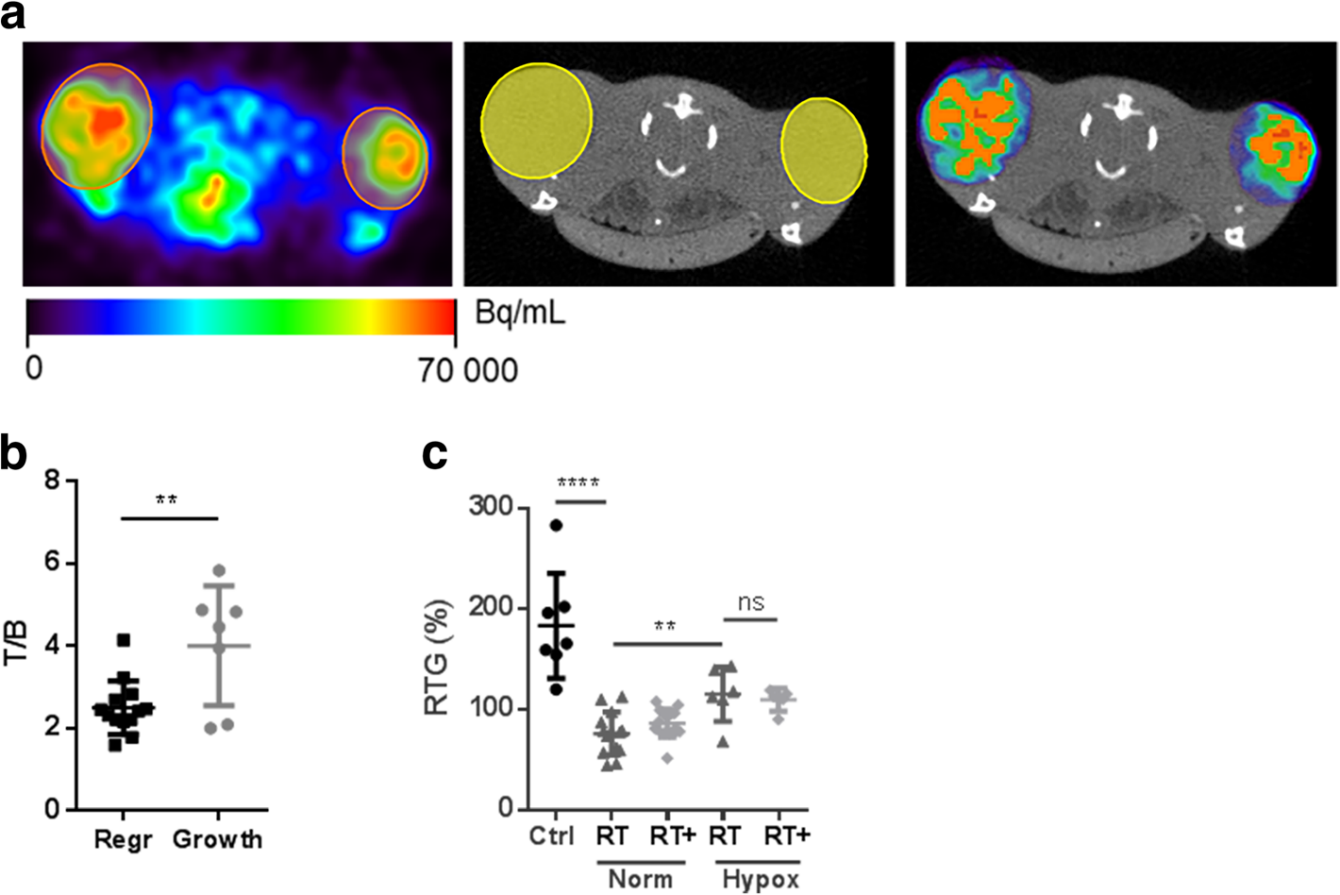
Predictive value of 18F-FAZA PET/CT in EAC xenografts. (**a**) Transverse slices at the level of the hind legs with mice in prone position. PET/CT acquired 3 h after tracer injection. Left: ^18^F-FAZA PET images with subcutaneous EAC tumors delineated spherically (orange). Middle: corresponding CT images. Right: Overlay ^18^F-FAZA PET/CT. The PET data exterior to the ROI’s was erased. Orange = ROI > 40% isocontour. High ^18^F-FAZA uptake was also seen in the urinary bladder due to renal excretion of the tracer. (**b**) Pre-treatment ^18^F-FAZA uptake of RT treated tumors. Regr = tumors that regressed (radiosensitive), Growth = tumors that continued growing (radioresistant). T/B ratios (single values, mean, SD, t-test). (**c**) Control (Ctrl); Radiotherapy (RT); Combination (RT+). Hypoxia status was defined by ^18^F-FAZA PET/CT: T/B < 3.59 = normoxic; T/B ≥ 3.59 = hypoxic. RTG of EAC xenografts (single values, mean, SD, t-test)

Forty-eight tumors were included for ^18^F-FAZA PET/CT (control *n* = 7, RT *n* = 20, combination *n* = 21) (Fig. 1a). T/B ratios were equally distributed across the treatment groups and varied from 1.17 to 5.83 with a median of 2.74. Tumors that regressed after RT (RTG < 100%) were defined radiosensitive (65%, *n* = 13) and tumors that continued growing (RTG > 100%), radioresistant (35%, n = 7). Pre-treatment ^18^F-FAZA uptake (T/B ratios) was significantly higher in radioresistant tumors than in radiosensitive tumors (*P* = 0.0046) (Fig. 1b), demonstrating that more hypoxic tumors are more resistant to RT than less hypoxic tumors. ROC-analysis was performed to identify a cut-off value for predicting RT response with ^18^F-FAZA PET/CT, which showed that a T/B of 3.59 predicted treatment response with the highest sensitivity and specificity (92.3% and 71.4% respectively, AUC 0.75). Based on pre-treatment ^18^F-FAZA PET/CT, tumors were divided in normoxic (T/B < 3.59) and hypoxic (T/B ≥ 3.59). Irradiation inhibited tumor growth significantly better in normoxic tumors compared to hypoxic tumors (*P* = 0.0025) (Fig. 1c).

### Hypoxic radioresistance and radiosensitizing effect of nimorazole

**Fig. 2:**
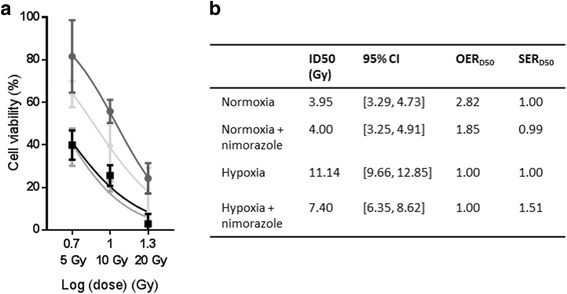
In vitro effects of nimorazole on radiotherapy (RT) response. (**a**) Normoxia;  Hypoxia;  Normoxia + nimorazole;  Hypoxia + nimorazole. Dose-response curve of MTS-assay with RT doses (x-axis, logarithmic) and cell viabilities (y-axis, mean, SD, non-linear regression fitted curve) relative to controls (0 Gy, cell viability = 100%). (**b**) ID50 = Radiation Dose (RD) to inhibit 50% of the cell viability; OER_D50_ = Oxygen Enhancement Ratio (RD hypoxia/normoxia); SER_D50_ = Sensitizer Enhancement Ratio (RD hypoxia/hypoxia with nimorazole)

First, radiosensitizing effects of nimorazole were investigated in vitro in the OACM5 1.C SC1 cell line (Fig. 2a-b). As expected, RT was less efficient under hypoxic conditions, illustrated by an upwards movement of the dose-response curve (OER_D50_ = 2.82). Pretreatment with nimorazole radiosensitized hypoxic tumor cells (SER_D50_ = 1.51). Nimorazole had no effect on RT efficacy in normoxic conditions and acted as a pure radiosensitizer without intrinsic cytotoxic effect (Additional file 1: Figure S1).

**Fig. 3:**
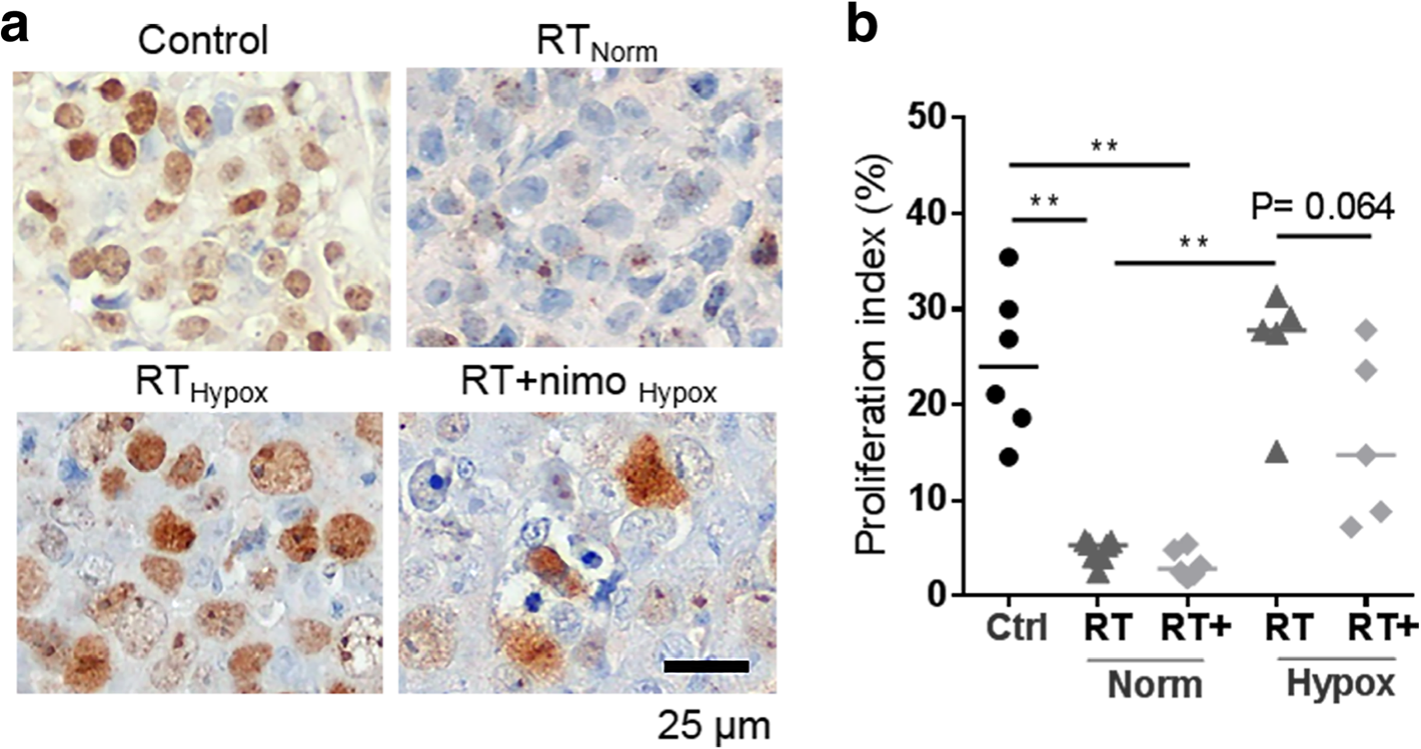
Effect of nimorazole on cancer cell proliferation in vivo. (**a**) Representative pictures of Ki67 stained tumor sections of each treatment group. Normoxic and hypoxic tumor areas were based on pimonidazole staining of consecutive sections. (**b**) Control (Ctrl); Radiotherapy (RT); Combination (RT+). Cancer cell proliferation indices from Ki67 staining (single values, median). Norm (normoxia) and Hypox (hypoxia) were based on pimonidazole staining of consecutive sections. (Mann-Whitney test)

Second, nimorazole was investigated in vivo. Histologic examination of EAC xenografts showed that hypoxic tumor areas were resistant to RT with significantly higher proliferation indices than in normoxic areas (*P* = 0.0025) (Fig. 3a, b). Pre-treatment with nimorazole radiosensitized hypoxic cancer cells with a trend to decrease proliferation indices (*P* = 0.064). Evaluation of the effect of nimorazole on tumor growth control showed that it had no effect in less hypoxic tumors (T/B < 3.59) (Fig. 1c). Further, opposite to the in vitro and histological results where nimorazole increased radiosensitivity in hypoxic conditions, nimorazole did not seem to improve tumor growth control in hypoxic tumors (T/B ≥ 3.59). (Fig. 1c).

## Discussion

This study investigated the predictive value of ^18^F-FAZA PET/CT for hypoxia-induced radioresistance and the radiosensitizing effect of nimorazole in an EAC model in mice. We showed that pre-treatment ^18^F-FAZA PET/CT could identify more and less hypoxic tumors, which was related with radiation response. We identified a T/B of ≥3.59 that predicted radioresistance with a sensitivity of 92.3% and specificity of 71.4%. Further, nimorazole clearly decreased hypoxia-induced radioresistance in the OACM5 1.C SC1 cell line in vitro and in the EAC xenografts (histologically). Moreover, this was the first study investigating ^18^F-FAZA PET in esophageal cancer. We focused on esophageal adenocarcinoma because it has become the main subtype in patients in the United States and Northern and Western Europe [28].

It is difficult to compare the T/B ratios of this study with others, because no consensus exists for quantifying ^18^F-FAZA uptake. Some studies quantify tracer uptake as percentage of the totally injected activity (%ID/g or SUV (standardized uptake values) if standardized to the animals’ weight). However, because FAZA is excreted in urine and feces, tracer activity at the moment of the scan can vary substantially between animals, making %ID/g or SUV parameters rather unreliable. Here, ^18^F-FAZA uptake was quantified relative to a reference non-hypoxic tissue (tumor to background ratio), according to a method used by Tran et al. [27] and was found feasible. For the future, it will be a challenge to use a uniform parameter.

Here, nimorazole was shown to have a SER_D50_ of 1.51 in hypoxic conditions in vitro, which is in accordance to previous literature [23, 29]. Also histologically, nimorazole increased radiation response in hypoxic tumor areas (Ki67 staining). The lack of its effect on tumor growth control could be for the following reasons. First, tumors were harvested one day post-treatment to evaluate the histological effects of radiation/nimorazole. This was rather early to analyze the total effect on tumor growth and we believe a longer follow-up could result in more significant differences. Second, single RT doses were used in vitro, whereas in vivo*,* more clinically relevant doses (5 × 5 Gy) were used. As it is known that fractionation causes tumor cell reoxygenation, the RT regimen itself could have radiosensitizing effects, minimizing the effect of nimorazole [30, 31]. Whether nimorazole will have a sensitizing effect in a clinical radiation regimen (23 × 1.8 Gy according to the recent CROSS trial [1]), is to be investigated.

As ^18^F-FAZA PET/CT has already been proven to be safe in the clinical setting, these results encourage a subsequent clinical trial where the predictive value of ^18^F-FAZA PET/CT is investigated in EAC patients. This could lead to a more patient-tailored approach. For example, if a tumor is predicted to show a good response, it seems to be worth to administer neoadjuvant treatment before surgery. Meanwhile, if a tumor is predicted to be resistant to neoadjuvant treatment, it could be better to perform the surgical resection earlier or to modify the neoadjuvant treatment and decrease radioresistance, like modifications to the RT regimen itself (e.g. dose-painting [32]) or addition of a hypoxia modifier (e.g. nimorazole [33]). Still, tumor hypoxia is distributed heterogeneously in space and over time [3]. For sure, repeating ^18^F-FAZA PET/CT scans will be needed to reevaluate tumor’s hypoxia status and indications for radiosensitizers.

Some limitations have to be taken in consideration regarding the present study. First, one tumor model (subcutaneous xenografts) was investigated with one tumor type (EAC), which limits conclusions and future clinical trials to this tumor type. The subcutaneous model was chosen because a previous study with orthotopic esophageal tumors localized at the distal esophagus was not feasible. Tumors could not be delineated due to background tracer activity in the liver (hepatic metabolization of FAZA). We believe visualization in patients will be better because of larger structures and higher soft tissue resolution on human CT scans. Further, this should not be a problem in esophageal squamous cell carcinomas that are typically located in the thoracic part of the esophagus. We believe that repeating the study at an orthotopic site is of little interest at the moment. By demonstrating the predictive value of ^18^F-FAZA in esophageal adenocarcinoma xenografts, we believe the next step should be a clinical study instead of another preclinical experiment. Second, because the cut-off T/B was defined retrospectively, the predictive value should ideally be confirmed in a prospective experiment. Third, other modification methods than nimorazole (e.g. dose-painting or carbogen breathing) could have been included to compare effects.

Tumor hypoxia is a long-known problem within oncology with little impact in the daily clinic. This is partially because hypoxia detection methods have not reached the routine clinical work-up of cancer patients. To continue enhancing patients’ outcomes and minimizing useless treatments, we are convinced a patient-tailored approach is required where tumor hypoxia will be one of the guiding biomarkers.

## Conclusions

This study showed that pre-treatment ^18^F-FAZA PET/CT is predictive for radiotherapy response in esophageal adenocarcinoma xenografts and encourages a subsequent clinical trial where the predictive value of ^18^F-FAZA PET/CT is investigated in esophageal adenocarcinoma patients. The benefit of the hypoxia modifier nimorazole was modest and asks for further investigation.

## Additional file

Additional file 1: **Figure S1**. In vitro effect of nimorazole monotherapy. Ctrl = control; Nimo = nimorazole; Norm = normoxia; Hypox = hypoxia. Y-axis shows absorbance, analyzed 72 h after treatment. No significant difference was observed between cells treated with PBS (control) or treated with nimorazole, under hypoxic or normoxic conditions (t-test). (DOCX 20 kb)

## Abbreviations

AUC: Area under the curve
CAIX: Carbonic anhydrase
EAC: Esophageal adenocarcinoma
EF5: Pentafluoropropylacetamide
FAZA: Fluoroazomycin arabinoside
FETNIM: Fluoroerythronitroimidazole
FFPE: Formalin-fixed paraffin-embeded
FMISO: Fluoromisonidazole
HIF: Hypoxia-inducible factor
HNSCC: Head and neck squamous cell carcinoma
IP: Intraperitoneal
OER: Oxygen enhancement ratio
RT: Radiotherapy
SER: Sensitizer enhancement ratio
T/B: Tumor to background
