# Supplementary material for: Hypoxia imaging with 18F-FAZA PET/CT predicts radiotherapy response in esophageal adenocarcinoma xenografts
Source: Radiat Oncol. 2018 Mar 7;13:39. doi: 10.1186/s13014-018-0984-3 (PMC5842657; doi:10.1186/s13014-018-0984-3)
Supplement: Supplementary file 1 — Figure S1. In vitro effect of nimorazole monotherapy. Ctrl = control; Nimo = nimorazole; Norm = normoxia; Hypox = hypoxia. Y-axis shows absorbance, analyzed 72 h after treatment. No significant difference was observed between cells treated with PBS (control) or treated with nimorazole, under hypoxic or normoxic conditions (t-test). (DOCX 20 kb) [file 13014_2018_984_MOESM1_ESM.docx]

# **Suppl. Fig. 1**


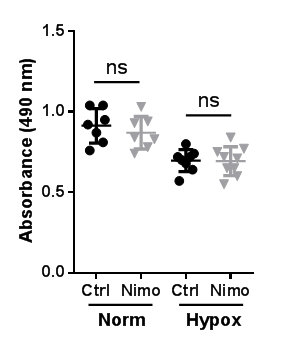


**Suppl. Fig. 1. *In vitro* effect of nimorazole monotherapy.**

Ctrl= control; Nimo= nimorazole; Norm= normoxia; Hypox= hypoxia. Y-axis shows absorbance, analyzed 72h after treatment. No significant difference was observed between cells treated with PBS (control) or treated with nimorazole, under hypoxic or normoxic conditions (t-test).
